# A Data-Driven Predictive Approach for Drug Delivery Using Machine Learning Techniques

**DOI:** 10.1371/journal.pone.0031724

**Published:** 2012-02-23

**Authors:** YuanYuan Li, Scott C. Lenaghan, Mingjun Zhang

**Affiliations:** Mechanical, Aerospace and Biomedical Engineering Department, University of Tennessee, Knoxville, Tennessee, United States of America; University of Minho, Portugal

## Abstract

In drug delivery, there is often a trade-off between effective killing of the pathogen, and harmful side effects associated with the treatment. Due to the difficulty in testing every dosing scenario experimentally, a computational approach will be helpful to assist with the prediction of effective drug delivery methods. In this paper, we have developed a data-driven predictive system, using machine learning techniques, to determine, *in silico*, the effectiveness of drug dosing. The system framework is scalable, autonomous, robust, and has the ability to predict the effectiveness of the current drug treatment and the subsequent drug-pathogen dynamics. The system consists of a dynamic model incorporating both the drug concentration and pathogen population into distinct states. These states are then analyzed using a temporal model to describe the drug-cell interactions over time. The dynamic drug-cell interactions are learned in an adaptive fashion and used to make sequential predictions on the effectiveness of the dosing strategy. Incorporated into the system is the ability to adjust the sensitivity and specificity of the learned models based on a threshold level determined by the operator for the specific application. As a proof-of-concept, the system was validated experimentally using the pathogen *Giardia lamblia* and the drug metronidazole *in vitro*.

## Introduction

A fundamental principle underlying effective treatment of a pathogen is the trade-off between rapidly curing the disease and preventing harm to the patient induced by the treatment. Currently, due to the expense associated with drug testing and the infeasibility of testing every possible dose experimentally, treatments are very difficult or impossible to optimize with respect to the balance between patient toxicity and pathogen killing. Another factor to consider when balancing this trade-off is mutation within the natural population, or specific drug induced mutation. The emergence of drug resistant strains of pathogens, including hepatitis B virus, methicillin resistant *Staphylococcus aureus* (MRSA), and tuberculosis, have had a dramatic impact on world health [1], [2], [3], [4]. The CDC estimates that 94,360 cases of MRSA occur each year in the U.S. alone resulting in 18,360 deaths [5]. The emergence of many drug resistant strains has been linked to patient non-compliance, due to undesirable side-effects associated with the treatment [6]. When the patient begins to feel better, they stop taking the drug so that side-effects can be avoided. This will allow the pathogen to recover and increase the potential for a drug resistant strain to emerge. In order to determine a more thorough approach to selecting the optimum dose, thus minimizing excessive drug, machine learning techniques were used to develop a framework for analyzing the trade-off between the drug dosage and effective pathogen killing.

The proposed system works as follows. First, the drug dose and cell population are discretized into distinct states. Then, the state changes are analyzed over time. After a Probabilistic Suffix Automaton (PSA) model makes sequential prediction of the drug-cell dynamics based on current observations, the future drug-cell dynamics are predicted given current observations of the drug dose and pathogen population. The combined framework can predict future drug-pathogen dynamics based on current observations and also determine the effectiveness of a drug delivery method based on the experimental training data. The overall framework was developed to meet the following criteria:

- Scalability – the system must be able to scale to multiple drugs and pathogen interactions. Due to the difficulty in scaling ordinary differential equations with multiple drugs [7], machine learning algorithms were employed.
- Minimum human supervision – the system must be able to learn and predict the drug-pathogen dynamics in an unsupervised, autonomous fashion. This will represent an improvement on other supervised, rule-based expert systems [8].
- Adaptivity – the system must be able to adapt to a constantly evolving pathogen population. As opposed to Hidden Markov Models, which are fixed after the initial training [9], a data-driven Variable Length Markov Model (VLMM) was used for increased flexibility.
- Online learning – the system must be able to interpret dynamic drug-pathogen sample points instead of dealing with a static dataset. Since offline learning algorithms such as Bayesian were not suitable [10], online learning algorithms were developed using a Fuzzy C-Mean (FCM) clustering technique and VLMM.

To validate the proposed system, we have used *Giardia lamblia* and metronidazole as a test case. *Giardia* is a protozoan parasite of the intestinal tract of mammals, reptiles, and birds, and the causative agent of giardiasis [11]. Currently, *Giardia* is responsible for the largest number of waterborne outbreaks of diarrhea in the United States and infects approximately 2% of adults and 6 to 8% of children in developed countries [12], [13]. The incidence of infection greatly increases in countries with poor water treatment facilities, and can lead to death in children in poverty stricken areas. Since the late 1950s, metronidazole and other nitroimidazoles have been used for treatment of giardiasis [14], [15]. However, drug resistance to metronidazole has been observed in humans, resulting in a failure of treatment [16]. As described earlier, the emergence of metronidazole resistant *Giardia* strains has been linked to the severity of side-effects, often lead to patients discontinuing the treatment after feeling better [17]. Since the mechanism leading to drug resistance in *Giardia* is patient non-compliance due to harsh side-effects, this serves as an ideal test case to validate the framework.

The goal of this research was to develop a methodology for an *in silico* approach to test multiple dosing options with a limited set of experimental data. Using *Giardia* as an *in vitro* test case, the cell count after various doses of metronidazole was used to generate both the training and testing data sets. Based on these data sets, a system was developed to classify the drug dosage and cell population into distinct classes, and predict the future states from current observations and effectiveness of treatment. While this study has focused only on *in vitro* applications, the method developed herein, has the potential to be extended to a clinical setting, to help doctors, with limited data, to predict if a current dosing method was effective or ineffective. This paper represents an important first step in the incorporation of machine learning technique to address the complex problem of pathogen drug resistance and effective therapy.

## Materials and Methods

### 1.1 System architecture

**Figure 1:**
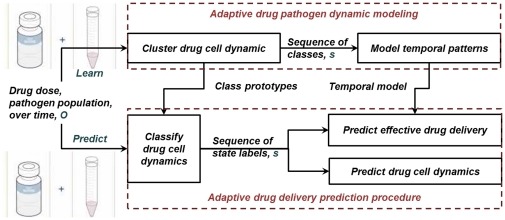
Diagram of the proposed machine learning procedure. The top part of the diagram shows the training procedure, which contains the clustering module and temporal analysis module. The bottom part shows the testing procedure.

**Figure 1** shows the architecture of the sequential drug delivery prediction framework. The drug concentration and pathogen population were used as a two-dimensional vector input. First the input was clustered into categories using a FCM clustering algorithm (described in **Section 1.2**). After categorization, the temporal patterns of the drug-pathogen dynamics were analyzed using a VLMM model that was implemented in the form of a PSA (described in **Section 1.3**). The system was able to make two kinds of predictions based on the PSA model: 1) the drug-pathogen dynamics over time, based on current observations (**Section 1.4**), and 2) the effectiveness of the drug using a likelihood-ratio detector (**Section 1.5**). In this paper, online, unsupervised learning was used since the pathogen was capable of evolving over time under selective pressures. All learning algorithms used were data-driven and adaptable.

### 1.2 Categorize drug-pathogen dynamics using an FCM clustering technique

Typically in the medical and drug delivery fields, FCM clustering algorithms have been used in analysis of medical imaging, including PET, MRI, and CAT scans [18], [19], [20]. However, in this study, an FCM clustering algorithm was used to categorize the drug concentration and the pathogen population into distinct categories. A major advantage of the FCM clustering technique is its fuzziness, in which a single data pattern may belong to several clusters, having different membership values in each cluster. This property could be advantageous when dealing with noisy or incomplete data, common in typical drug delivery applications. In this paper, an FCM clustering algorithm was developed similar to Dunn's [21], which was improved upon by Bezdek [22]. The algorithm is described as follows.

Given a set of 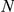 data observations, i.e., 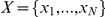, and a number of desired clusters 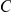. The FCM clustering algorithm minimizes the following objective function:
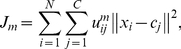
(1)where 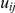 is the degree of membership of 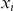 in the cluster 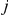. 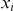 is the *i*-th *D*-dimensional input vector, 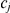 is the prototype of the center of cluster 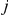, 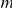 is a weighting exponent on each fuzzy membership such that 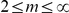, and 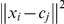 is a distance measurement between the data 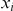 and cluster center 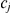. The objective function 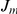 is minimized via an iterative process. The update function for the cluster centers 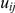 is defined as follows:
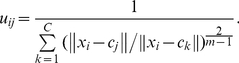
(2)The update function for the cluster center prototype 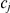 is defined as follows:
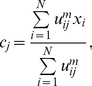
(3)where 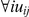 satisfies: 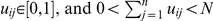. Iteration will stop, when 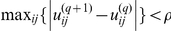, where 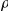 is a termination criterion between 0 and 1, whereas 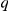 is the iteration step. This procedure converges to a local minimum or a saddle point of 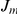.

In terms of the drug delivery system, 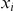 represents the combination of the cell population and drug concentration at each observation point for 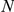 number of observations. For example, if there are 24 trials in the training dataset, with 10 observation points in each trial, then 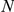 is equal to 240. From these 240 points, natural clusters, groups of similar observations, were identified. The total number of observations is then classified into individual states, 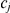, from 1 to 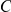, where 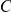 is the total number of desired states. The value of 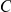 can be selected in collaboration with experimental researchers to yield a set of states that have biological meaning. For example, if the goal was to evaluate effective and non-effective only, then 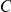 would equal 2. However, for applications that require diverse analysis, 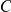 could be set to a higher value. The Fuzzy function was used to increase the robustness of the clustering procedure due to potentially noisy experimental data. This function allows for partial membership in multiple clusters, instead of forcing membership to only one cluster. After the training procedure, the state prototypes are obtained, and the prototypes are used to analyze the testing data. Note that the FCM technique allows the model to scale to multiple drugs by increase the dimensionality of 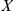. By incorporating multiple drug concentrations with pathogen populations into clustering models, each cluster can then represent the multi-drug effects on pathogen population change.

In the testing procedure, the drug-pathogen observations, *O*, are categorized during the prediction phase, described as follows: given 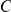 distinct category/state prototypes, denoted by 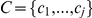, learned during the training phase, and a series of 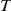
observations 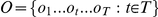 made over time, where *t* denotes the time index, the system finds the closest match of the current observation 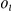
to a state prototype in 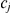. The formulation is defined as follows,
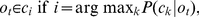
(4)where 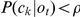, which means that the difference between 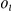
and the closest match 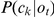
is less than a vigilance parameter 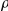. If this is the case, then the drug-pathogen observation 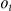 is categorized as 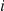. If the difference is larger than 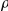, then a new drug-pathogen dynamic state will be generated. If we let 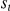 denote the state label learned from observation 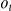, at time 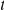, then there is a one-to-one mapping from observations to state labels. Thus, a sequence of observations 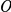, becomes a sequence of states 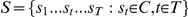.

Using the methods described above, it was possible to categorize the observations of the cell-drug interactions into clearly defined states. These states contain information about the cell population and drug concentration at each observation point, but contain no temporal information. To analyze the temporal pattern associated with the changes between states, it was necessary to use another technique to obtain this information.

### 1.3 Analyze temporal pattern using VLMM model

As described above, FCM categorizes the data into state sequences, which are often temporally correlated. To analyze these temporal dependencies, a Markov chain model was used. Since Markov models have difficulty in capturing higher level dependencies in the sequence data, we chose to use a high order (memory) Markov model. For a large set of time-correlated sequential data, statistical correlations decrease rapidly with increasing distance between symbols in the sequence. If the statistical correlations are indeed decreasing, then there exists a *memory* length 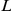 such that the empirical probability changes very little if conditioned on subsequences longer than 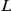. In other words, the memory length depends on the context and is not fixed. In this study, a VLMM model [23] was used to preserve the minimal subsequences (of variable lengths) that are necessary for precise modeling of the given statistical source. This results in a more flexible and efficient sequence representation.

In addition, a symbolic predictive PSA model was constructed in this work to interpret and predict data based on the drug/pathogen dynamics. In the PSA model, the continuous time observations were first abstracted to a discrete space, analogous to a set of finite states. Symbolic modeling and processing have several advantages over continuous measurements and models in drug-pathogen dynamics, including: 1) discrete states have clear physical meaning and are easy for humans to interpret, e.g., drug effective state; and 2) are less impacted from noise, while still preserving the essential underlying pattern or dependencies that govern the behavior in the observed domain. These advantages of the PSA model make it more suitable to model temporal sequences in drug-pathogen dynamics, compared to other models.

In general, each state in a PSA is labeled by a string of state sequences over a finite alphabet 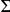. The transition function between the states is defined based on these state sequence labels. Given a state sequence 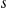, the algorithm traverses the underlying graph of the PSA, and ends in a state labeled by a suffix of the sequence. When a PSA generates a state sequence, the probability distribution of the next symbol generated is defined given the previously generated subsequence of length, at most, 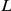. Therefore, the probability distributions these automata generate can be equivalently generated by Markov chains of order 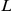. However, since the size of order-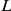 Markov chains is exponential in 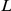, their estimation requires a data length and time exponential in 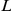.

For any drug delivery trial, the state at each observation point is combined to form the state sequence. 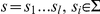, the longest suffix of 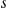 can be denoted as 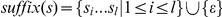. The PSA 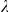 is a 5-tuple, 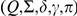, consisting of

- a finite set of states 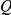 (e.g., natural states of drug-pathogen interactions),
- a finite alphabet 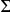, in this scenario, the number associated with each state (e.g., 1,2,3,…)
- a transition function 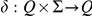, which describes the transition from one state to another. For example the transition from a high drug, high pathogen state to a low drug, low pathogen state.
- the next symbol probability function 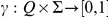,
- and an initial probability distribution over the starting states 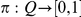.

**Figure 2:**
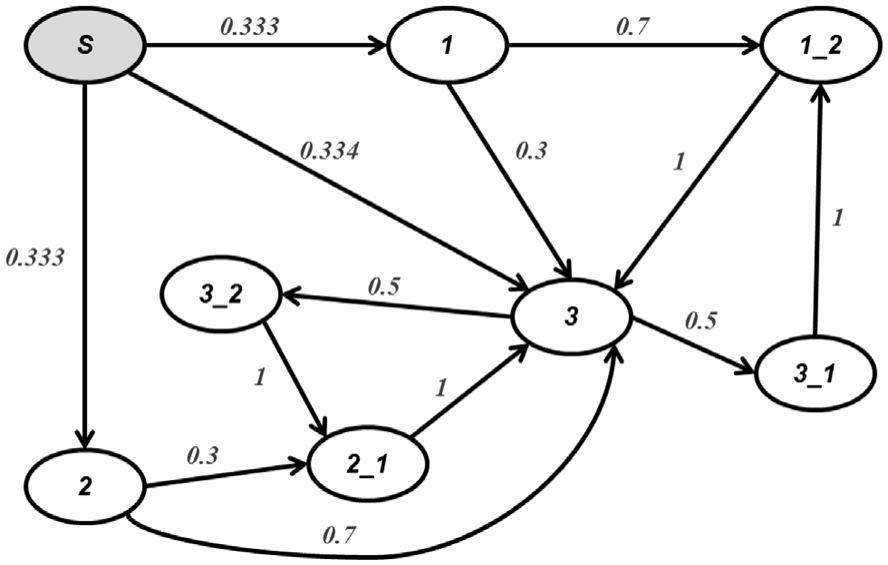
Example of second order PSA built from the state sequence, 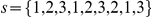. This state transition diagram shows the probability of transitioning between states. The sum of all possible states transitions equals one, indicating that a state transition must occur.

Note that the function 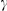 and 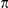 must satisfy the following conditions for every 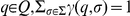 and 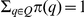. These equations maintain that the probability of transitioning between all possible states is 1, as graphically represented in **Figure 2**. This means that there is a 100% probability that some transition will occur.

For any given 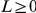, *L*-PSA is a Markov chain of order 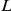. It is a process satisfying 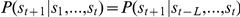, where 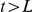. We define the empirical probability of a sub-sequence 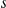, over the given sample set, as the number of times that the subsequence was observed in the sample set divided by the maximal number of (possibly overlapping) occurrences of a pattern of the same length. The conditional empirical probability of observing a state 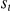 right after a given sub-state sequence is defined as the number of times that the state symbol appears after the given subsequence divided by the total number of times that the sub-sequence has appeared, followed by any state symbol. Specifically, the frequencies 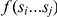
of a sub-state sequence 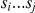 in the state sequences 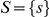 is given by 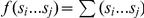, where 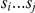 is a sub-sequence of 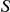. The conditional empirical probability of observing the state 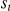 right after the state sequence 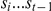 is defined by 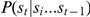, which can be estimated using sub-state sequence frequencies as follows:
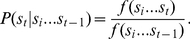
(5)Note that this leads to a natural definition of a probability distribution over all state sequences of length 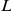 since 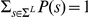.

As an example, consider a drug-pathogen state sequence 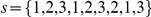 with a three letter alphabet 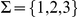. **Figure 2** shows the second-order PSA learned from the sequence. The example PSA has eight possible states, namely, “Start”, “1”, “2”, “3”, “1_2”, “2_1”, “3_1”, and “3_2”. Note that “Start”, “1”, and “2” are transient states; “3”, “1_2”, “2_1”, “3_1”, and “3_2” are recurrent states. Detailed information on PSA construction and inference procedures can be found in [23].

To model the normal behavior using the Maximum Likelihood criterion, a model was developed that maximizes the probability of a given sequence of observations. Specifically, we let 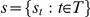 denote a temporal sequence of size 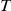, where 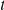 denotes the discrete sampling time. Given a PSA 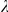, the total likelihood of the observations can be expressed mathematically as 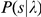. Matching the drug concentration and pathogen population states against a suffix automaton yields the cumulative probability of the given state sequence being generated by the PSA. To match a sequence against a PSA, the algorithm starts with the first state symbol and finds its corresponding state in the automaton. Next the model traverses the sequence appending the next state symbol onto the previous one. The algorithm then finds the current state based on the newly created state label. If the node exists, the probability of transitioning from the previous state to the current state is noted, and the algorithm continues appending state symbols. If the node does not exist, however, one symbol at a time is removed from the front of the state label until a node is encountered that does exist in the automaton. In the worst case, the program will have to backtrack to a transient node, since it should contain the next-symbol probability, in order to proceed with the matching algorithm. In general terms, the PSA searches the string of states for observations that have occurred previously. If a state has previously occurred, then a new state is not created, however, if the PSA searches the string and finds no instances of the current state in the previous states, then a new state is created.

The system can use the learned PSA model to predict the drug-pathogen dynamics based on the current state 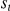. The details of the prediction are discussed in **Section 1.4**. If the probability of the observation sequence given the model is below a threshold 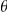, obtained from the Receiver Operating Characteristic (ROC) curve as described in detail in **Section 2.4**, then the drug delivery method is categorized as ineffective. A likelihood-ratio verification scheme has been created and is addressed in detail in **Section 1.5**.

### 1.4. Predict drug-pathogen dynamic using VLMM model

Given the drug-pathogen interaction state sequence, 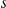 its prediction by a PSA model 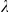 can be conducted state by state, where the cumulative probability in the sequence is calculated by transitioning from one state to another, in a chainlike manner. The problem can be formulated as follows: given a history, i.e., the 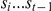 sequence of 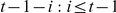 drug-pathogen interaction states in the past, the method predicts the next drug-pathogen dynamic state. The approach achieves the goal by finding the state 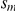 that maximizes the posterior probability of state 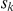. Formally it is defined as follows,
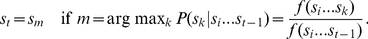
(6)


It is desirable to predict several consecutive drug-pathogen dynamic states in a set window. A prediction window, of length 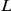, is defined as the prediction of a drug-pathogen state sequence 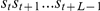 knowing its history 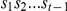. The system can achieve this by interactively predicting the next state and appending the existing state sequence up to length 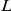 based on past history.

Through the above approach, it will be possible to predict the outcome of doses based on past sequence information. It will also be possible to predict doses that have not been experimentally tested as described in **Section 2.2**.

### 1.5 Predict drug delivery effectiveness using likelihood ratio-based verification

To predict the effectiveness of a drug delivery strategy, a likelihood-ratio based prediction method is used. In this example, we assume that there is a training period, consisting of different drug delivery methods in the training dataset. The effective drug delivery methods are used to build a normal model, whereas variations from the effective model are treated as an ineffective drug delivery method. In this case we let 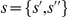 denote the entire drug delivery dataset, and 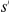 denote the normal training drug delivery state sequences, while 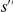 denotes the target testing drug delivery state sequence. Given a sequence(s) of normal training drug delivery trial(s) 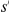, and a sequence of testing drug delivery states 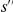, the task of verifying the effectiveness of a drug delivery method can be formulated to determine if 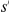
is the same as 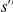. Note that if the effective drug delivery methods are not available, the random variable 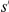
can represent an ineffective drug delivery method, and an effective drug delivery method can be detected when 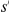 matches 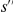. The drug delivery verification problem is formulated in the same manner. The task can be restated as a hypothesis test between:
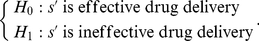
(7)The likelihood-ratio test to decide between these two hypotheses is given by:
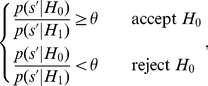
(8)where 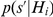, 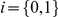, is the probability density function for the hypothesis 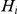 evaluated for the observed sequence 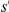, also referred to as the likelihood of the hypothesis 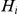 given the sequence. The decision threshold for accepting or rejecting 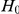 is 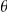. For our drug delivery verification, the *null* and *alternative* hypothesis use PSA models 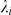. Hence, we denote the PSA model for the *null* hypothesis as 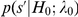 and for the alternative hypothesis as 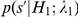. The likelihood-ratio sequential verification process is given by 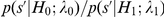. Typically, the logarithm of this statistic is used giving the log-likelihood ratio 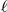,
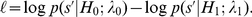
(9)


During the training period, various drug delivery trials were encountered that were effective, ineffective and/or undetermined. The model for 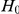 was estimated using an effective event sequence. However, the model for 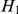 was less well defined since it must represent every ineffective drug delivery method. Since it is too expensive to run every ineffective drug trial, it is not possible to train 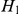 with every ineffective drug delivery method. Therefore, all hypothesized sequences of events, was a more suitable way to define 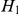.

### 1.6 System evaluation, sensitivity and specificity analysis

The following performance metrics were used to evaluate the drug delivery verification system: True Positive Rate (*TPR*), True Negative Rate (*TNR*), False Positive Rate (FPR), and False Negative Rate (*FNR*). These performance metrics can be visualized using a matrix. The matrix is often referred to as confusion matrix (shown in **Table 1**). Ideally, the values of sensitivity and specificity are at 100%; the values of false alarm rate and miss rate are at 0%.

**Table 1:** Performance metrics.

		Condition (Gold standard)	
		*Positive*	*Negative*
Prediction outcome	*Positive*	True Positive	False Positive
	*Negative*	False Negative	True Negative
		Sensitivity = TP/(TP+FN)	Specificity = TN/(FP+TN)

Typical performance metrics table that reports the number of True Negatives, False Positives, False Negatives, True Positives, Sensitivity and Specificity.

The sensitivity and specificity of a diagnostic test depends on more than just the “quality” of the test – they also depend on the definition of what constitutes an abnormal test. In practice, we choose a threshold 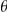 above which we consider the test to be abnormal and below which we consider the test to be normal. The position of the threshold will determine the number of true positive, true negatives, false positives and false negatives. Different thresholds can be used for different clinical situations if the goal is to minimize one of the erroneous types of test results. A Receiver Operating Characteristic curve was used to a guideline to balance the tradeoff between sensitivity and specificity.

### 1.7 Experimental setup

*Giardia lamblia* trophozoites were obtained from the American Type Culture Collection, strain designation 30957. Trophozoites were grown and maintained anaerobically in modified Keister's media at pH 7.0, limiting cyst formation [24]. Eight doses, 0 µg/ml, 1.9 µg/ml, 6.19 µg/ml, 10.95 µg/ml, 16.67 µg/ml, 20.4 µg/ml, 32 µg/ml, and 50 µg/ml, were tested to provide a range of killing curves. Prior to the addition of drug, 10^5^ cells/ml were added to 2 ml glass screw cap tubes and incubated for 30 minutes in a 37°C incubator. After this adjustment period, the drug was added at time point 0 to the tubes and the cells were placed back in the incubator. At 0, 4, 10, and 18 hours the cell number was counted using a hemocytometer to determine the number of cells/ml. This is a typical *in vitro* method used to determine the effectiveness of drugs on *G. lamblia*, and has previously been validated for determination of metronidazole activity specifically [25], [26], [27], [28], [29]. All experiments were conducted in triplicate. The half-life of metronidazole was estimated based on previous pharmacokinetic data [30], [31], [32].

## Results and Discussion

### 2.1 Experimental results

**Figure 3:**
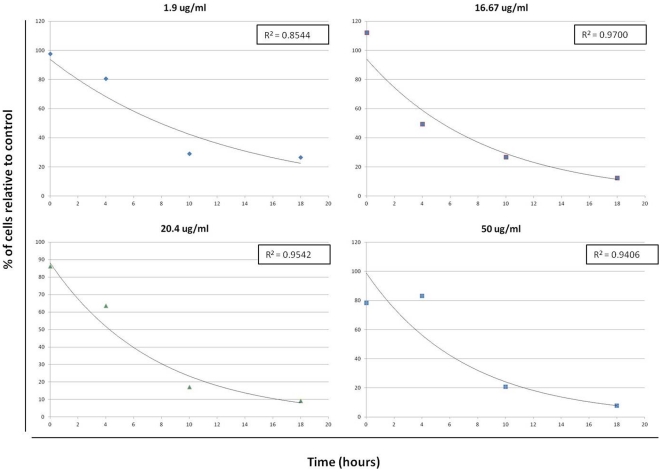
Representative *Giardia* killing curves. Killing curves generated from the average of three replicates per dose. The cell counts were compared to the control, which is typical of killing curves. Note that the general trend is towards an exponential decrease over the time interval of the study, 18 hours. In the lowest dose tested, 1.9 µg/ml, an obvious plateau is reached at 18 hours indicating an ineffective dosing, while at the other doses, the trend indicates a decline.

Killing curves were generated from the averaging of the three replicates from each dose, examples of which are shown in **Figure 3** with the numerical data displayed in **Table 2**. In addition to these curves, the data from each trial was plotted and the points connected to illustrate the trend from each set of doses (**Figure S1**). From this data, it was determined that the ineffective doses were doses where the population at 18 hours was greater than the starting population. In these curves, the cell population was normalized to the starting population, i.e., starting cell number/current cell number. Using this normalization, increases in the cell number over time will have values greater than 100%, whereas decreasing populations will have numbers less than 100%. In terms of the biological implication of this normalization, if the replication of trophozoites occurs every 8–12 hours [33], then if the cell population is at the 100% threshold after 18 hours, when the drug has significantly decayed, the population will continue to increase and eventually reach a maximum population level. At the initial time points, between 0 and 4 hours, all trials, including the controls, exhibited a decline of similar proportions. This decline was most likely due to a temperature effect that resulted in a sampling error. Similarly, all trials displayed an increase in population from 4 to 10 hours, although the degree of growth varied. Since the effect of the drug is not immediate, and the cell cycle was not synchronized, it was not surprising that a delay in killing was observed in this study. An advantage of the system developed in this study is that the system acts only to compare the trends observed, allowing analysis of potentially misleading data at the early stages. After acquiring the data from the experimental studies, the data were preprocessed for incorporation into the system for analysis and prediction.

**Table 2:** Cell number over time for each dose prior to pre-processing.

	Time interval (hours)			
Dose (µg/ml)	*1*	*4*	*10*	*18*
***0***	1×10^5^	6.4×10^4^	4.1×10^5^	5×10^5^
***1.9***	1×10^5^	5.1×10^4^	1.2×10^5^	1.3×10^5^
***6.19***	8.5×10^4^	3.9×10^4^	1.6×10^5^	7.2×10^4^
***10.95***	1.2×10^5^	6.2×10^4^	1.5×10^5^	3.9×10^4^
***16.67***	1.1×10^5^	3.2×10^4^	1.1×10^5^	6.2×10^4^
***20.4***	8.9×10^4^	4.1×10^4^	7.1×10^4^	4.5×10^4^
***32***	9×10^4^	5.1×10^4^	1.2×10^5^	3.9×10^4^
***50***	8.1×10^4^	5.3×10^4^	8.6×10^4^	3.9×10^4^

The average cell count for each time interval and dose is displayed in the chart above in cells/ml. As indicated in the methods, the starting cell concentration was 1×10^5^ cells, however, variations always arise when making counts with a hemocytometer. This data was used to generate the dose curves shown in **Figure 3**.

### 2.2 Preprocessing

As described above, four sampling points were collected for each drug trial and a killing curve was generated for each trial. From each curve, data points were extrapolated for 2, 6, 8, 12, 14, and 16 hours. In addition, the drug concentrations were normalized based on the highest dose of 50 µg/ml administered. The drug concentrations at each sampling point were estimated using a 6 hour half-life for metronidazole [30], [31]. The drug-pathogen dynamics were classified into 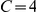 states. Hence, the state sequence 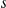 had an alphabet size of 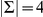. The physical meaning of the 4 states are defined as follows:

- current pathogen population (48∼100%) with high (24∼50 µg/ml) drug concentration,
- current pathogen population (13∼171%) with a medium (7.65∼20.4 µg/ml) drug concentration,
- current pathogen population (28∼229%) with low (0∼6.84 µg/ml) drug concentration,
- and current pathogen population (267∼702%) with low (0∼3.42 µg/ml) drug concentration.

Note that depending on the application, the user can choose the desired number of states by setting the parameter 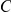 in the FCM clustering algorithm.

**Figure 4:**
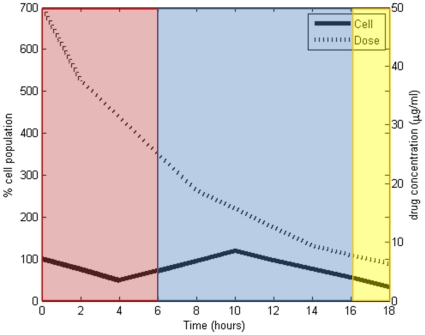
An example of 50 µg/ml trial and its states. The separated boxes indicate different clusters/states. The width of the boxes indicates the time duration of each state. The dotted curve represents the drug concentration over time, while the solid curve represents the percent change of the pathogen over time.

**Figure 5:**
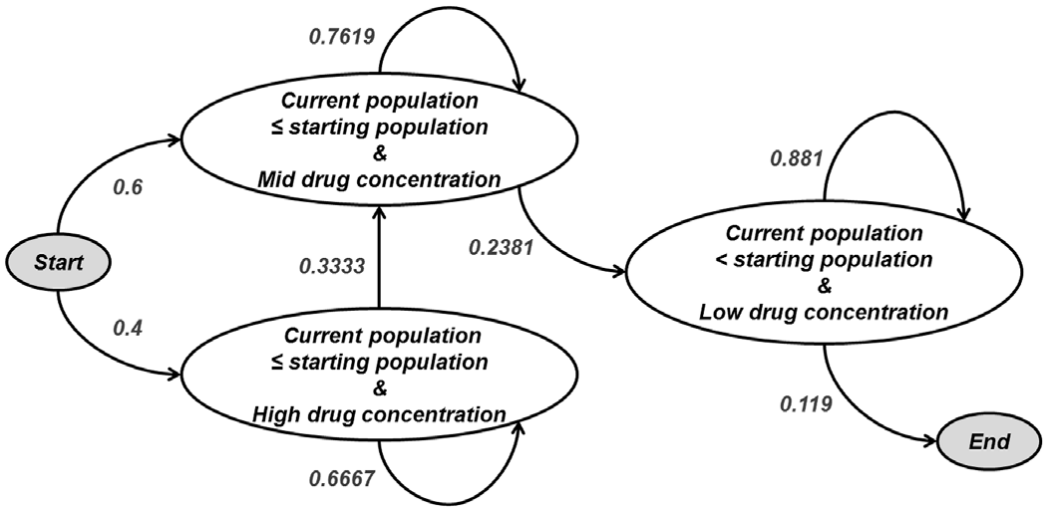
The Markov model state transition diagram built from the 15 effective drug delivery trials. The “start” and “end” states are added for illustrative purposes. In the effective delivery strategy, it is possible to transition between three states. From the high drug state it is only possible to remain in that state, or transition to the medium drug state. Similarly, once in the medium drug state it is not possible to transition back to the high drug state, it is only possible to remain in that state or transition to the low drug state. Once in the low drug state, the system will remain in the state for various iterations before finally ending.

**Figure 6:**
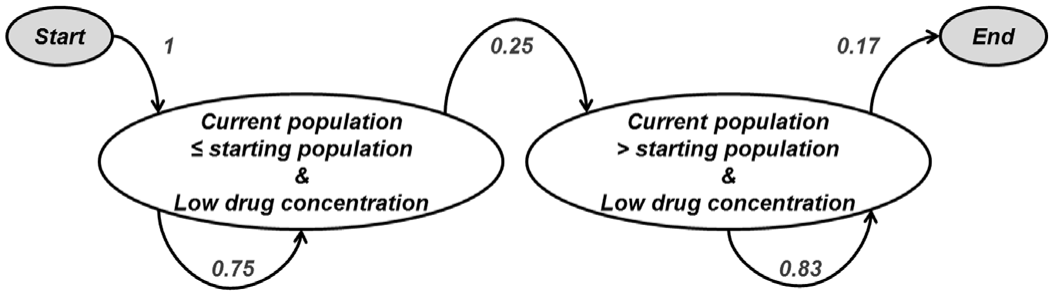
The Markov model state transition diagram built from an ineffective drug delivery. The “start” and “end” states are added for illustrative purpose. In this example of ineffective delivery, the model has only two transition states. When the concentration of drug is low and the current population is below the starting population, the system is more likely to remain in this state for several iterations. Eventually, however, a transition out of this state will occur resulting in a low drug concentration and a larger current population. Once in this state it is impossible to leave this state, and eventually an end state will be reached.

**Figure 4** displays an effective drug delivery method. The drug concentration and cell population were categorized by the Fuzzy C-Mean clustering algorithm as indicated by the colored boxes in **Figure 4**. As illustrated in the figure, this trial started with a high drug concentration and cell population of 100%, and transitioned to a state with medium drug concentration and cell population between 12∼171%, and finally transitioned to the effective dosing end state of low drug concentration and cell population from 28–229%. Since the effective dosing all terminated in the same state, a PSA was constructed for effective dosing using all effective trials, **Figure 5**. In contrast, the ineffective dosing showed various transitions between the states, and did not always terminate in the same state. For this reason the PSAs for ineffective dosing (1.9 µg/ml, 6.19 µg/ml and 10.95 µg/ml) were created by combining all three trials of that dose. As an example, a PSA was constructed based on the training data and an ineffective drug delivery method combining all three trials at 1.9 µg/ml, shown in **Figure 6**. It should be noted that the PSAs were built autonomously using the developed system, and that the “start” and “end” states were added for illustrative purposes only. As illustrated in **Figure 5** and **Figure 6** **,** the effective and ineffective drug delivery methods show different temporal patterns. Based on analysis of the data, 15 trials with effective dosing and 9 trials with ineffective dosing were identified. Since, the overall goal of the study was to use the learned PSA models to predict the drug-pathogen dynamics and predict the effectiveness of an unknown drug delivery method; the following experiments were conducted to demonstrate this application and validate the developed model for realistic drug delivery data.

### 2.3 Drug-pathogen dynamics prediction

In order to predict the drug-pathogen dynamics, the system uses the Partial Prediction Matching (PPM) procedure, equation (6), on the PSA model and the partial test sequence. To verify the ability of the system to predict future drug-pathogen states, a 3-fold cross-validation method was used on the available drug trials. As stated in the experimental setup, three drug trials were conducted for each dosing method, resulting in 24 individual trials. For each round of cross validation, the 24 trials were partitioned into two groups: training set and testing set. The training set contained eight trials with one trial from each of the eight doses. The testing set contained the other sixteen trials. To reduce variability, multiple rounds of cross-validation were performed using different partitions and the validation results were averaged over the rounds. **Table 3** shows the averaged prediction accuracies of 3-fold cross validation for different partial matching sequence lengths. The 3-fold cross validation for all trials was averaged to determine the accuracy of the prediction at each observed sequence length. As the observation sequence grew, the prediction became more accurate, e.g., when the system received one observation, the prediction accuracy was 53.33%; while when the system received nine observations, the prediction accuracy increased to 97.5%. Based on the available drug delivery trials, the system had an accuracy greater than 73.33% when the observation length was ≥4. It should be noted that the more data points that are generated, the greater prediction efficiency over time. The results from a Student's T-test, assuming normal distribution, comparing the accuracy of all observation lengths, i.e. observation length 1 vs. 2, 1 vs. 3, etc., confirmed that the difference in results were statistically significant (99.5% confidence interval).

**Table 3:** The prediction performance of 3-fold cross-validation for all doses.

*Observed sequence length*	*1*	*2*	*3*	*4*	*5*	*6*	*7*	*8*	*9*
*Accuracy (Mean)*	53.33	60.83	67.08	73.33	79.58	85.0	90.0	93.75	97.50
*Accuracy (Variance)*	8.91	8.25	7.1	6.44	4.72	3.1	2.0	1.79	0.08

The averaged prediction accuracies for variable sequence lengths for all dosing methods. (control, 1.9 µg/ml, 6.19 µg/ml, 10.95 µg/ml, 16.67 µg/ml, 20.4 µg/ml, 32 µg/ml, and 50 µg/ml).

**Table 4** shows the average prediction accuracies of 3-fold cross-validation of drug delivery trials for each dosing method with different partial matching sequence lengths. The average accuracy for each observed sequence length was obtained from the average of 3-fold partitions of drug delivery trials. The prediction accuracies for each dosing method followed the same trend – as the observation lengths grew, the system was able to predict more accurately. This experiment also showed that some dosing methods were easier to predict then others, e.g., a drug dose of 1.9 µg/ml had a prediction accuracy of 10% when only one observation was available, while dose 6.29 µg/ml had a prediction accuracy of 100% when only one observation was available. This phenomenon was related to the complexity of the trend. Trends with distinct features were easier to predict than ones that were not as distinct.

**Table 4:** The average prediction accuracies of 3-fold cross-validation for each dose.

*Observed sequence length*	*1*	*2*	*3*	*4*	*5*	*6*	*7*	*8*	*9*
***Control***	40.0	40.0	40.0	40.0	50.0	60.0	70.0	80.0	90.0
***1.90*** ** µg/ml**	10.0	20.0	30.0	40.0	50.0	60.0	70.0	80.0	90.0
***6.19*** ** µg/ml**	100.0	100.0	100.0	100.0	100.0	100.0	100.0	100.0	100.0
***10.95*** ** µg/ml**	86.7	96.7	96.7	96.7	100.0	100.0	100.0	100.0	100.0
***16.67*** ** µg/ml**	70.0	80.0	90.0	100.0	100.0	100.0	100.0	100.0	100.0
***20.40*** ** µg/ml**	60.0	70.0	80.0	90.0	100.0	100.0	100.0	100.0	100.0
***32.00*** ** µg/ml**	40.0	50.0	60.0	70.0	80.0	90.0	100.0	100.0	100.0
***50.00*** ** µg/ml**	20.0	30.0	40.0	50.0	60.0	70.0	80.0	90.0	100.0

The prediction accuracy based on sequence length shows a similar trend observed when all of the curves were averaged. As the sequence length increases for each does, the prediction accuracy increases. The only exception is the dose of 6.19 µg/ml, where the accuracy is 100% because this dose does not transition out of the initial state. In cases with more complex transitions, a larger sequence length is needed for accurate predictions.

The previous experiments demonstrate the system scalability when all dosing methods are available. In practice, however, it is not possible to test all drug dosing schemes. In a second experiment, the training data contained five drug delivery methods (control, 6.19 µg/ml, 16.67 µg/ml, 32 µg/ml, 50 µg/ml): two ineffective dosing methods (control, 6.19 µg/ml) and three effective drug delivery methods (16.67 µg/ml, 32 µg/ml, 50 µg/ml). After completion of the training phase, three doses (1.9 µg/ml, 10.95 µg/ml, and 20.4 µg/ml), were evaluated. It should be noted that the only data available to the model was the initial dose and starting cell population, the sequences generated from the experimental data were not available, and only used for subsequent validation. For example in testing dose one (1.9 µg/ml), the only state given to the model was the initial state 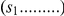. The PSA then predicted the other 9 states based on this initial state 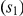. Next, the model was given two states 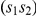 from the experimental data 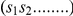
and the other 8 states were predicted by the PSA. These iterations continued until all 10 states from the experimental system were input into the model. To evaluate the accuracy of the prediction of the PSA given variable state lengths, the predicted state sequence was compared to the experimental state sequence. **Table 5** shows the prediction accuracy based on the starting number of states given to the model. As the observed sequence length increased, our system was able to make more accurate predictions. It was determined that the system needed at least four observations to have an accuracy of ∼74%, based on the training dataset and partial observations. The prediction accuracy of the system was 53% when only one observation was given and 98% when eight observations were given. Results from a student's T-test again found that all results were significant with a confidence level of 99.5%. The strong significance of data with both analysis techniques indicates that the system is able to accurately predict the future drug-pathogen states, given an adequate sequence length.

**Table 5:** The prediction performance for *in silico* dosing.

*Observed sequence length*	*1*	*2*	*3*	*4*	*5*	*6*	*7*	*8*	*9*
*% Accuracy (Mean)*	52.5	60.0	67.5	73.8	80.0	85.0	90.0	93.8	97.5
*% Accuracy (Variance)*	9.4	8.6	8.2	7.4	5.4	3.4	2.0	0.8	0.2

Average prediction accuracies based on the training data sets (control, 6.19 µg/ml, 16.67 µg/ml, 32 µg/ml, 50 µg/ml) and the testing data sets (1.9 µg/ml, 10.95 µg/ml, and 20.4 µg/ml).

In the context of the *Giardia* testing case that was used in this paper, if experimental researchers were able to generate four observations, the model would be able to accurately predict the rest of the state sequences ∼74% of the time. This means that with the training data used (control, 6.19 µg/ml, 16.67 µg/ml, 32 µg/ml, 50 µg/ml) it would be possible to predict the effectiveness of all treatments within this range, given these four initial points. When trying to determine the trade-off between the effectiveness of treatment while reducing the concentration of drug, this would provide a complete set of data for mitigating this balance. Experimentally testing all of these doses would be impossible, but can quickly be achieved *in silico* using the system developed in this study.

### 2.4 Model validation for prediction performance

To predict the effectiveness of a drug delivery trial, a likelihood ratio-based verification technique was used, as defined in Equation (9). In order to verify the performance of the prediction procedure, the following procedure was used: during the training period, five drug trials (control, 10.95 µg/ml, 32 µg/ml, 50 µg/ml) to obtain the alternative hypothesis 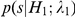, and the three effective drug delivery methods (10.95 µg/ml, 32 µg/ml, 50 µg/ml) to obtain the null hypothesis 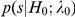. The learned universal PSA model 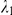 consists of 80 nodes and the learned drug effective PSA model 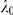 consists of 60 nodes. Two effective drug trials (16.67 µg/ml, 20.4 µg/ml) and two ineffective trials (1.9 µg/ml, and 6.19 µg/ml) were used for testing the proposed prediction system. Each drug trial was completed over 18 hours, and the data were expanded to include 10 observation points. For each drug delivery method, the state sequence length was incrementally increased to the drug trial length 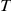, where 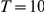, e.g., 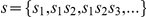 and the log likelihood-ratios were obtained for the tested drug-pathogen state sequences, i.e., 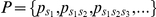.

**Figure 7:**
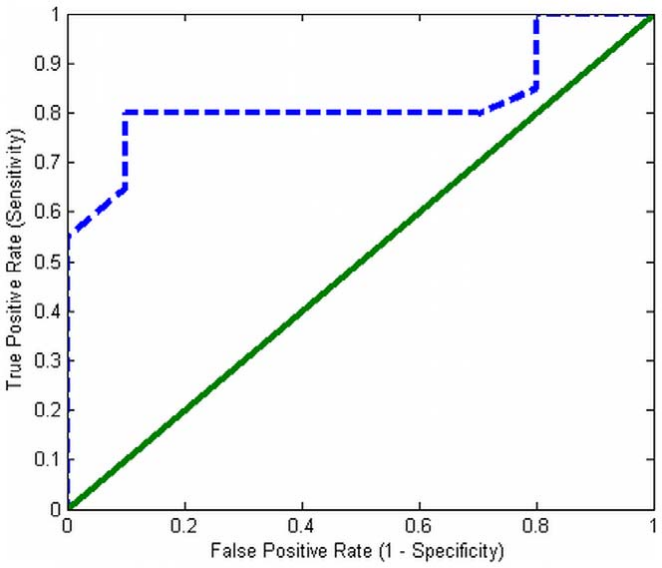
The ROC curve for determining prediction performance. The ROC curve shows the tradeoff between sensitivity and specificity (any increase in sensitivity will be accompanied by a decrease in specificity). The closer the curve is to the minimum false alarm rate (*x*-axis) and the maximum sensitivity (*y*-axis), the more accurate the test. As the ROC curve approaches *y = x*, the less accurate the test becomes. The intersection point of the ROC curve with the line *y = −x* is defined as the optimum operation point. In this ROC curve, the optimum operation point had an 80% true positive rate, with a 20% false positive rate.

In order to determine the verification threshold 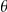, a Receiver Operating Characteristic curve was used. The ROC curve plots the *TPR*/sensitivity vs. the *FPR*/false alarm rate for a binary verification (2-class) system as its verification threshold is varied. **Figure 7** shows the ROC curve constructed from the testing trials. Note that each prediction result (or one instance of a confusion matrix) represents one point in the ROC space. The best possible prediction has no false negatives or false positives. From **Figure 7**, it was observed that the optimal operating point for the proposed verification system has a *TPR* of 80% and a *FPR* of 20%. This optimal operating point (*TPR* = 80%, *FPR* = 20%) was used as the reference to set the likelihood-ratio threshold 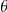 value in Equation (8). **Table 6** shows the sensitivities and specificities obtained when adjusting the threshold parameter 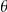. The threshold value of 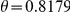 gives the optimal trade-off between sensitivity and specificity in our drug delivery test environment. As mentioned previously, depending on the application, different threshold values can be used to meet the user requirements. In the current test case, the AUC for the generated ROC curve was found to be 85%. The current experimental setup has validated that the developed system is able to predict the effectiveness of unseen drug delivery methods with good performance (accuracy ∼85%). In addition, the system is adaptable to multiple applications. For example, if the drug delivery task requires high specificity (i.e., ∼100%) with low sensitivity (i.e., ∼50%), the system can adjust the threshold to 1.25. In this way the system is robust for a variety of applications, and can be tuned by an operator.

**Table 6:** Thresholds for different trade-offs.

*Threshold* 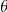	*Sensitivity*	*1-Specificity*
**…**	…	…
**0.8179**	0.8000	0.2000
**0.8601**	0.8000	0.3000
**0.8864**	0.8000	0.4000
**0.9044**	0.8000	0.5000
**…**	…	…
**1.2462**	0.5000	1.0000
**…**	…	…

Different sensitivities and specificities of the prediction system by adjusting threshold values. For example, if a threshold of 0.8179 is selected, then the sensitivity predicts performance would be 80% and the rate of getting the false alarm rate is 20%.

In summary, the developed system is able to predict the future drug-pathogen dynamics based on current observations. Our experimental results have shown that the prediction performance is able achieve 74% when four observations are made. In addition, our developed system is able to predict the effectiveness of a drug dosing method *in silico* based on the current observations of the environment with high performances. Our experimental results have shown that they system is able to obtain an overall accuracy of 85%. In addition, the system is able to achieve 80% of true positive rate and 20% of false positive rate for the optimal trade-off point. Depends on the application, users can adjust the threshold to achieve higher true positive rate or false positive rate. The learning system is able to cluster the drug-pathogen dynamics into discrete states and analysis the temporal dependencies among the state in a fully autonomous fashion.

### 2.5 Conclusion

In this paper, we have developed a machine learning framework that models the drug-pathogen dynamics. The framework can be used to test the outcome of a variety of doses given a limited number of experimental data. The proposed framework has been validated *in vitro* through experimental study with *Giardia lamblia*. Based on the framework, the system first learns to categorize the drug-pathogen interactions into a discrete set of states using a Fuzzy C-Mean clustering algorithm. It then uses a PSA to model the temporal state sequences. The learned models can be used to predict the drug-pathogen dynamics based on the past history. In addition, a likelihood-ratio verification method was used to predict the effectiveness of a given drug delivery method. Due to the experimental limitations, the data collected is often noisy and incomplete. Therefore, online, unsupervised, data-driven methods were chosen. This proposed method was validated experimentally for the drug/cell interaction between *Giardia lamblia* and metronidazole. Using the method, it was possible to predict the dynamic drug-cell states over time, and the effectiveness of the treatment strategy. The accuracy of prediction increased from 73% with four data points to 97.5% with nine data points. Performance evaluation of the system, when predicting the effectiveness of the strategy, revealed an accuracy of 85%, using an ROC method. This system can predict the effectiveness of multiple dosing schemes, allowing for reduced experimental costs, and an increased speed of prediction. This will allow researchers to investigate more effective treatment options by evaluating a larger pool of doses than possible with experiments. It will also be possible for a user to adjust the treatment, if the current treatment has been determined to be ineffective. In this way it will be possible to monitor patient non-compliance by monitoring the drug dose and pathogen population on a return visit to determine the effectiveness of the treatment. Future work will use a Markov Decision Process to separate the dose into discrete actions, based on the observations of pathogen population and side effects, the system will then decide to increase or decrease the dosage. Throughout this process an optimum dosing strategy could be learned, balancing the trade-off between side-effects, pathogen population, and the dosage.

## Supporting Information

Figure S1 ***Giardia*** **killing trends from various doses of metronidazole.** The *Giardia* staring population was normalized to 100%. The *Giardia* cells were counted at hours 0, 4, 10, and 18 and were normalized based on control. Eight doses were used, 0 µg/ml, 1.9 µg/ml, 6.19 µg/ml, 10.95 µg/ml, 16.67 µg/ml, 20.4 µg/ml, 32 µg/ml, and 50 µg/ml. Three drug delivery trials were conducted for each dose.
(TIF)
