# Supplementary figures and images for: A Data-Driven Predictive Approach for Drug Delivery Using Machine Learning Techniques
Source: PLoS One. 2012 Feb 23;7(2):e31724. doi: 10.1371/journal.pone.0031724 (PMC3285649; doi:10.1371/journal.pone.0031724)

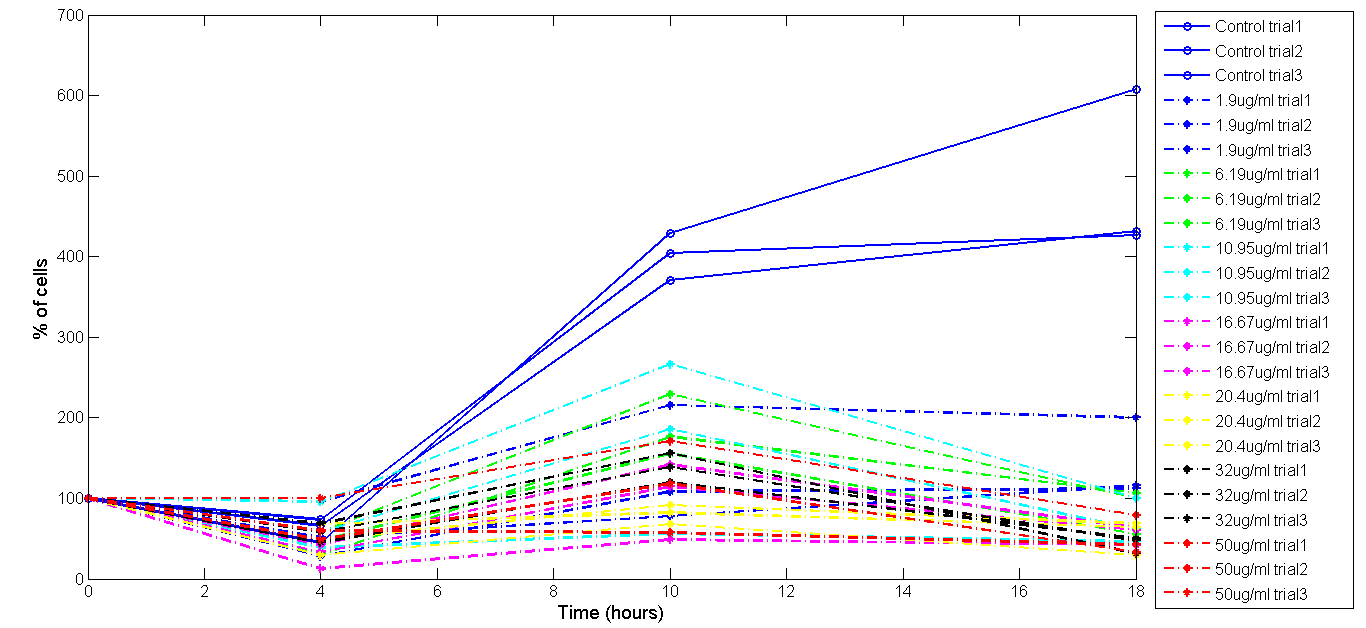

Supplement: Figure S1 — Giardia killing trends from various doses of metronidazole. The Giardia staring population was normalized to 100%. The Giardia cells were counted at hours 0, 4, 10, and 18 and were normalized based on control. Eight doses were used, 0 µg/ml, 1.9 µg/ml, 6.19 µg/ml, 10.95 µg/ml, 16.67 µg/ml, 20.4 µg/ml, 32 µg/ml, and 50 µg/ml. Three drug delivery trials were conducted for each dose. (TIF) [file pone.0031724.s001.tif]
